# Identifying and Extracting Rare Diseases and Their Phenotypes with Large Language Models

**DOI:** 10.1007/s41666-023-00155-0

**Published:** 2024-01-05

**Authors:** Cathy Shyr, Yan Hu, Lisa Bastarache, Alex Cheng, Rizwan Hamid, Paul Harris, Hua Xu

**Affiliations:** 1 Department of Biomedical Informatics, Vanderbilt University Medical Center, Nashville, TN 37203 USA; 2 School of Biomedical Informatics, University of Texas Health Science Center at Houston, Houston, TX 77225 USA; 3 Division of Medical Genetics and Genomic Medicine, Vanderbilt University Medical Center, Nashville, TN 37203 USA; 4 Department of Biostatistics, Vanderbilt University Medical Center, Nashville, TN 37203 USA; 5 Department of Biomedical Engineering, Vanderbilt University Medical Center, 2525 West End Avenue, Nashville, TN 37203 USA; 6 Section of Biomedical Informatics and Data Science, Yale School of Medicine, 100 College Street, New Haven, CT 06510 USA

**Keywords:** Natural language processing, ChatGPT, Rare disease, Artificial intelligence, Prompt learning, Large language model

## Abstract

**Purpose:**

Phenotyping is critical for informing rare disease diagnosis and treatment, but disease phenotypes are often embedded in unstructured text. While natural language processing (NLP) can automate extraction, a major bottleneck is developing annotated corpora. Recently, prompt learning with large language models (LLMs) has been shown to lead to generalizable results without any (zero-shot) or few annotated samples (few-shot), but none have explored this for rare diseases. Our work is the first to study prompt learning for identifying and extracting rare disease phenotypes in the zero- and few-shot settings.

**Methods:**

We compared the performance of prompt learning with ChatGPT and fine-tuning with BioClinicalBERT. We engineered novel prompts for ChatGPT to identify and extract rare diseases and their phenotypes (e.g., diseases, symptoms, and signs), established a benchmark for evaluating its performance, and conducted an in-depth error analysis.

**Results:**

Overall, fine-tuning BioClinicalBERT resulted in higher performance (F1 of 0.689) than ChatGPT (F1 of 0.472 and 0.610 in the zero- and few-shot settings, respectively). However, ChatGPT achieved higher accuracy for rare diseases and signs in the one-shot setting (F1 of 0.778 and 0.725). Conversational, sentence-based prompts generally achieved higher accuracy than structured lists.

**Conclusion:**

Prompt learning using ChatGPT has the potential to match or outperform fine-tuning BioClinicalBERT at extracting rare diseases and signs with just one annotated sample. Given its accessibility, ChatGPT could be leveraged to extract these entities without relying on a large, annotated corpus. While LLMs can support rare disease phenotyping, researchers should critically evaluate model outputs to ensure phenotyping accuracy.

## Introduction

Rare diseases are chronically debilitating, often life-limiting conditions that affect 300 million individuals worldwide [1]. Though individually rare (defined as affecting fewer than 200,000 individuals in the United States), rare diseases are collectively common and represent a serious public health concern [2]. Because of the lack of knowledge and effective treatment options for rare diseases, patients undergo diagnostic and therapeutic odysseys that have devastating medical, psychosocial, and economic consequences for patients and families, resulting in irreversible disease progression, physical suffering, emotional turmoil, and ongoing high medical costs [3–5]. Thus, there is an urgent need to shorten rare disease odysseys, and reaching this goal requires effective diagnostic and treatment strategies.

Phenotyping is crucial for informing both strategies and is a cornerstone of the study on rare diseases. Ongoing initiatives like the National Institutes of Health’s Undiagnosed Diseases Network rely on deep phenotyping to generate candidate diseases for diagnosis, identify additional patients with similar clinical manifestations, and personalize treatment or disease management strategies [6, 7]. In addition, phenotyping can facilitate cohort identification and recruitment for clinical trials critical to the development of novel treatment regimes [8, 9]. Rare disease phenotypes are often embedded in unstructured text and require manual extraction by highly trained experts, which is laborious, costly, and susceptible to bias depending on the clinician’s background and training. An alternative is to leverage natural language processing (NLP) models, which have the potential to automatically identify and extract rare disease entities, reduce manual workload, and improve phenotyping efficiency.

Automatic recognition of disease entities, or named entity recognition (NER), is an NLP task that involves the identification and categorization of disease information from unstructured text. This task is especially challenging due to the diversity, complexity, and specificity of rare diseases and their phenotypes, which can have different synonyms (e.g., neurofibromatosis type I and Von Recklinghausen’s Disease), abbreviations (e.g., NF1 for neurofibromatosis type I), and modifiers such as body location (e.g., small holes in front of the ear) and severity (e.g., extreme nearsightedness). Descriptions of rare disease phenotypes that are discontinuous, nested, or overlapping present additional challenges; moreover, those that range from short phrases in layman’s terms (e.g., distention of the kidney) to medical jargon (e.g., hydronephrosis) may further complicate NER.

While early approaches for NER relied on rules derived from extensive manual analysis, advancements in technology led to the emergence of large language models (LLMs), artificial intelligence systems built using deep learning techniques [10]. Specifically, LLMs use a deep neural network architecture called transformers that enable models to learn complex language patterns, capture long-range dependencies, and generate coherent responses [11]. LLMs are the bedrock of two major NER paradigms: 1) *pre-train and fine-tuning* and 2) *pre-train and prompt learning*. We henceforth refer to these paradigms as *fine-tuning* and *prompt learning*, respectively. The former involves a two-step process where a language model is first trained on a massive amount of unlabeled text data and then fine-tuned on specific downstream NER tasks with labeled data. In contrast, prompt learning is a more recent paradigm that reformulates the NER task as textual prompts so that the model itself *learns to predict the desired output* in the second step.

While fine-tuning LLMs has been shown to achieve strong performance on benchmark datasets [12], a major bottleneck is the development of large, annotated corpora. Recently, OpenAI released ChatGPT, a revolutionary LLM capable of following complex prompts and generating high-quality responses without any annotated data (zero-shot) or with just a few examples (few-shot) [13–16]. This capability, which provides opportunities to significantly reduce the manual burden of annotation without sacrificing model performance, is especially attractive for NER in the context of rare diseases. While some explored the potential of ChatGPT for diagnosing rare diseases with human-provided suggestions [17, 18], none have studied its performance for NER in the zero- or few-shot settings.

To this end, our study makes the following contributions. 1) This work is the first to explore prompt learning for biomedical NER in the context of rare diseases. Specifically, we designed new prompts for ChatGPT to extract rare diseases and their phenotypes (i.e., diseases, symptoms, and signs) in the zero- and few-shot settings. 2) We established a benchmark for evaluating ChatGPT’s NER performance on a high-quality corpus of annotated descriptions on rare diseases [19]. In addition, we compared prompt learning to fine-tuning by training and evaluating BioClinicalBERT, a domain-specific Bidirectional Encoder Representations from Transformers (BERT) model, on the annotated corpus [20]. 3) We conducted an in-depth error analysis to elucidate ChatGPT’s performance and 4) provided suggestions to help guide future work on prompt learning for rare diseases.

## Literature Review

Despite the proliferation of studies on NLP over the past decade, the task of NER is relatively under-explored for rare diseases. In this section, we provide a summary of prior contributions specific to extracting rare diseases and their phenotypes from unstructured text. These contributions can be broadly divided into two categories based on the NLP approach: 1) rule-based and 2) deep learning. Among those in the second category, only one explored fine-tuning [21]; to the best of our knowledge, none have explored prompt learning for rare disease NER to date.

Using rule-based algorithms, Davis et al. [22] identified individuals with multiple sclerosis from clinical notes in electronic health records (EHR). The authors manually reviewed patient notes to determine relevant keywords on disease progression and type, which were then used to build rule-based algorithms. For example, the algorithm for identifying the year of initial neurological symptom selected 100 characters around phrases referencing the beginning of the disease course, i.e., “dating back" and “began". Lo et al. [23] extracted phenotypes related to Dravet syndrome from clinical notes using the Unified Medical Language System Metathesaurus’ subset of 20,000 phenotypic words or expressions. Deisseroth et al. [24] developed ClinPhen, a rule-based phenotype extractor for genetic diseases that automatically converts clinical notes into a prioritized list of patient phenotypes using Human Phenotype Ontology terms. Nigwekar et al. [25] used an unnamed NLP software to identify patients with the terms “calciphylaxis" or “calcific uremic arteriolopathy" in their medical records.

Recently, Fabregat et al. [26] and Segura-Bedmar et al. [21] leveraged deep learning techniques, including bidirectional long short term memory (BiLSTM) networks and BERT-based models, to recognize rare diseases and their clinical manifestations from biomedical texts. Fabregat et al.’s BiLSTM model is a recurrent neural network that sequentially processes the input text from both forward and backward directions, allowing the model to learn contextual information on both sides. In their work, Segura-Bedmar et al. explored a similar model architecture and found that using a conditional random field (CRF) as the output layer led to improved performance. In addition, the authors trained domain-specific BERT models by fine-tuning them on the downstream NER task. Overall, fine-tuning BERT models had the highest accuracy, outperforming both BiLSTM and BiLSTM with a CRF layer.

## Methods

### Problem Definition

**Fig. 1:**
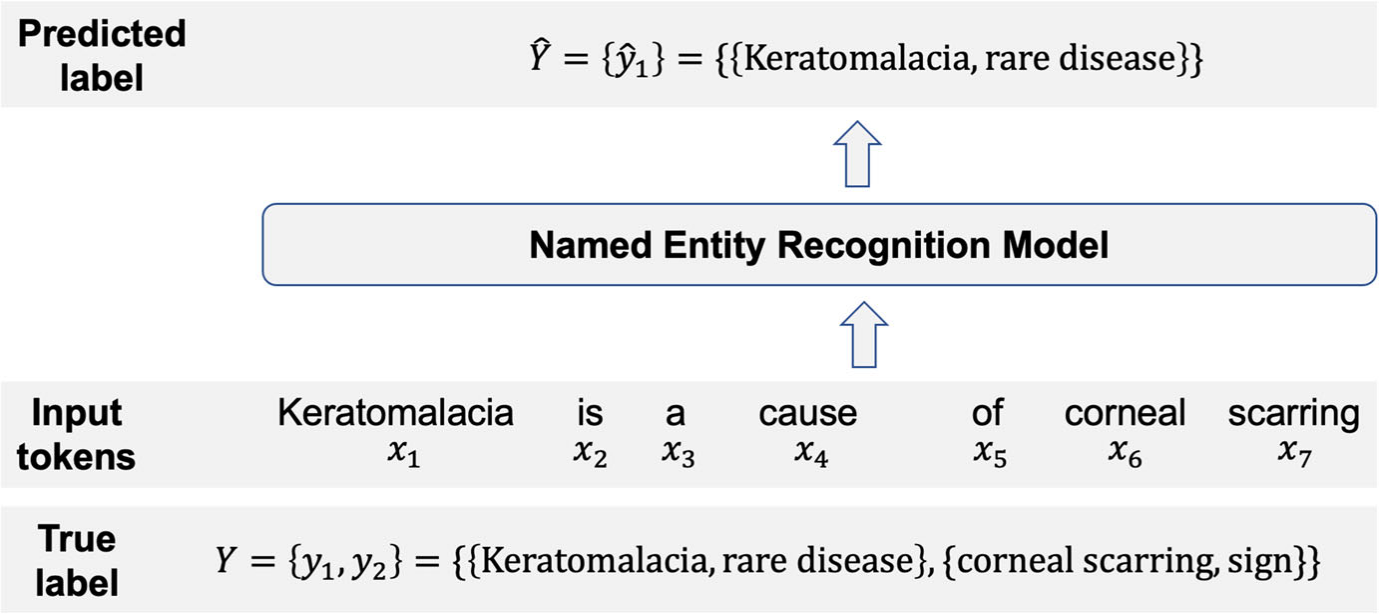
Example of the rare disease named entity recognition task. $\{x_i\}_{i=1}^7$ denotes the sequence of input tokens, $Y=\{y_1,y_2\}$ the true labels with $m=2$ entities, and $\hat{Y}=\{\hat{y}_1\}$ the predicted label with $\hat{m}=1$ entity

Our objective is to identify and extract rare disease-related named entities, which are words or phrases that belong to the pre-defined categories: rare disease, disease, symptom, or sign. As such, we seek to build an NER model that classifies each input token into a pre-defined category. Formally, given a sequence of *n* input tokens $X = \{x_1, x_2, \ldots , x_n\}$, the true label (i.e., gold-standard annotation) is the vector $Y := \{y_1, y_2, \ldots , y_m\}$ where

$$\begin{aligned} y_j = \{x_{\text {start}_j}:x_{\text {end}_j}, t_j\}, \quad 0 \le j \le m \le n \end{aligned}$$

is the tuple for the *j*th entity. Here, $\text {start}_j \in [1, n]$ and $\text {end}_j \in [1, n]$ denote the starting and ending indices of the *j*th entity, respectively, where $\text {start}_j \le \text {end}_j$ and $t_j \in \{\text {rare disease, disease, symptom, sign}\}$ is the entity type. We let

$$\begin{aligned} x_{\text {start}_j}:x_{\text {end}_j}= [x_{\text {start}_j} x_{\text {start}_{j+1}} \cdots x_{\text {end}_{j-1}} x_{\text {end}_j}] \end{aligned}$$

denote the textual span from $x_{\text {start}_j}$ to $x_{\text {end}_j}$ and let $\hat{Y} := \{\hat{y}_1, \hat{y}_2, \ldots , \hat{y}_{\hat{m}}\}$ denote the model-predicted label vector where

$$\begin{aligned} \hat{y}_k = \{x_{\hat{\text {start}}_k}: x_{\hat{\text {end}}_k}, \hat{t}_k\}, \quad 0 \le k \le \hat{m} \le n \end{aligned}$$

is the tuple for the *k*th predicted entity. Figure 1 shows an example where an NER model recognizes one of two named entities from the input, “Keratomalacia is a cause of corneal scarring." Here, the model correctly identified the rare disease, “keratomalacia," but missed “corneal scarring" as a sign.

### Dataset

To study the NER performance of LLMs for rare diseases, we used the RareDis corpus, which consists of $n = 832$ texts containing descriptions of rare diseases from the National Organization for Rare Disorders database [19]. This corpus was annotated with four entities (rare disease, disease, symptom, and sign) by biomedical experts who had an inter-annotator agreement (IAA) F1-score of 83.5% under exact match, indicating a high level of annotation consistency and reliability. Specifically, the F1-score measures the IAA accounting for precision (proportion of correctly annotated entities) and recall (proportion of gold-standard entities that were annotated). Table 1 provides the entity definitions and summary statistics. Unlike corpora with distinct entity types, e.g., {person, location, organization} or {problem, test, treatment}, RareDis consists of entities with considerable semantic overlap. Specifically, rare diseases are a subset of diseases. Diseases can cause or be associated with other diseases as a symptom or sign. The distinction between symptoms and signs is very subtle; while both are abnormalities that may indicate a disease, the former are subjective to the patient and cannot be measured by tests or observed by physicians (e.g., pain or loss of appetite). On the other hand, a sign can be measured or observed (e.g., high blood pressure, poor lung function). Across $n = 832$ texts, there were a total 4,065 rare diseases, 1,814 diseases, 316 symptoms, and 3,317 signs. Rare diseases and signs were more common than diseases and symptoms, accounting for 77% of all entities in the corpus (Table 1). A subset of the RareDis corpus (832 out of 1041 texts) is publicly available and distributed in the Brat standoff format [27].

**Table 1:** Entity definitions and summary statistics

Entity	Definition	Examples	Total	Count Per Text
			Count	Mean (SD)
Rare disease	Diseases which affect a small number of people	cat eye syndrome,	4,065	4.88 (3.57)
	compared to the general population	Marfan syndrome		
Disease	An abnormal condition of a part, organ, or system	cancer, cardiovascular	1,814	2.18 (2.59)
	of an organism resulting from various causes, such	disease		
	as infection, inflammation, environmental factors,			
	or genetic defect, and characterized by an identifiable			
	group of signs, symptoms, or both			
Symptom	A physical or mental problem that may indicate	fatigue, pain	316	0.38 (1.23)
	a disease or condition; cannot be seen and do not			
	show up on medical tests			
Sign	A physical or mental problem that may indicate	rash, abnormal heart	3,317	3.98 (4.89)
	a disease or condition; can be seen and shows up	rate		
	on medical tests			

Total count represents the number of entity occurrence in the entire corpus. SD = standard deviation

### NER Paradigms

In this section, we describe our approach to performing NER with LLMs under two paradigms: 1) fine-tuning and 2) prompt learning.

#### Fine-tuning BERT-Based Model

For fine-tuning, we chose BERT as our LLM for two reasons. First, BERT is one of the most widely-used deep contextualized language models, achieving state-of-the-art performance on benchmark NER datasets [12]. Specifically, its transformer architecture captures long-range dependencies in the input text and supports parallel processing, thereby enabling contextualized learning and reducing computational burden. Second, Segura-Bedmar et al. [21] found that fine-tuning BERT models resulted in the best NER performance on the RareDis corpus. Therefore, we adopted the same approach for a consistent comparison.

**Fig. 2:**
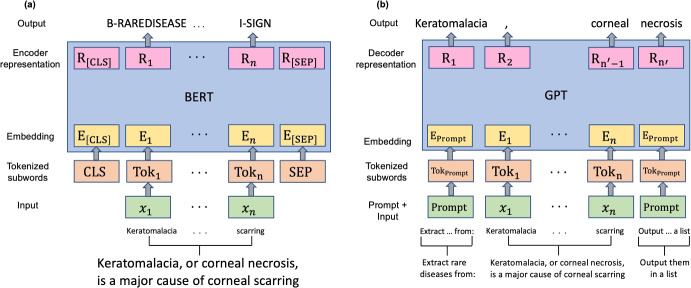
Architecture of **(a)** BERT and **(b)** GPT. $\{x_i\}_{i=1}^n$ denotes the sequence of input tokens. CLS and SEP are special tokens that represent classification and separation, respectively. *E* and *R* denote embeddings and representations, respectively

Figure 2 illustrates the architecture of the BERT model. To fine-tune this model on the RareDis corpus, we performed a series of pre-processing tasks. First, we split the texts into tokens with the BERT tokenizer and added special tokens (i.e., CLS and SEP) to the beginning and end of each tokenized sequence, respectively. Next, we converted the tokens to their respective IDs and padded (or truncated) text sequences based on the maximum number of tokens (i.e., 512) that a BERT-based model can handle, and created an attention mask to distinguish between actual and padding tokens. Last, we mapped the entity labels, {rare disease, disease, symptom, sign}, to corresponding numerical values. We partitioned the data into a training, validation, and test set based on an 8:1:1 ratio. For the base architecture, we selected BioClinicalBERT [20], a variant of BERT that was pre-trained on large-scale biomedical (PubMed, ClinicalTrials.gov) and clinical corpora (MIMIC-III [28]). The model fine-tuning parameters were learning rate = 2e-5, weight decay rate = 0.1, batch size = 32, and dropout = 0.1. BioCinicalBERT takes as input the sequence of tokens and produces context-based embeddings. These embeddings are then passed through a stack of transformer encoder layers that capture bidirectional, contextual information from each token. The layers output contextualized representations of the tokens, which are used to produce a probability distribution over output labels. Specifically, we used BIO (beginning, inside, outside) tags to represent the output labels, where B-*t* and I-*t* correspond to the first token and continuation of an entity mention of type $t \in \{\text {rare disease, disease, symptom, sign}\}$, respectively, and O for other tokens. Figure 2a shows an example where “Keratomalacia" and “scarring" were labeled B-rare disease and I-sign, respectively.

The BIO tags directly correspond to the model-predicted label vector defined in Section 3.1. For predicted entity $k < \hat{m}$ with type $\hat{t}_k$, the predicted starting token $x_{\hat{\text {start}}_k}$ corresponds to the input token with a B- $\hat{t}_k$ tag, and the predicted ending token $x_{\hat{\text {end}}_k}$ corresponds to the last input token with an I- $\hat{t}_k$ tag before the next token with a B- $\hat{t}_{k+1}$ tag, where $\hat{t}_k$ may or may not be the same as $\hat{t}_{k+1}$. For example, the *k*th predicted entity has type $\hat{t}_k = \text {rare disease}$, whereas the $k+1$st has type $\hat{t}_{k+1} = \text {sign}$. If $k = \hat{m},$ i.e., the *k*th predicted entity is the last mention in the input sequence, then the predicted ending token $x_{\hat{\text {end}}_k}$ is the last input token with an I- $\hat{t}_k$ tag.

#### Prompt Learning Using GPT-Based Model

In this section, we describe our approach to reformulating NER as a text generation task in the zero- and few-shot settings using OpenAI’s ChatGPT (GPT-3.5 turbo). The former refers to instructing the model to extract entities directly from an input text in the test set, and the latter is similar except we also provide an example of extracted entities from a training text. All experiments were performed using OpenAI’s application programming interface with the model gpt-3.5-turbo on June 19th and 20th, 2023. We used a temperature of 0 so that ChatGPT always selected the most likely token in its response to ensure reproducibility.

**Table 2:** Summary of prompts

Different parts of the prompt are color-coded as follows:  ,  ,  ,  , and  .  and  represent the entity and corresponding definition from Table 1

*Prompt design*. Table 2 provides a summary of prompts in the zero- and few-shot settings. The five main building blocks of our prompt designs were 1) task instruction, 2) task guidance, 3) output specification, 4) output retrieval, and, in the few-shot setting, 5) a specific example. Task instruction conveys the overall set of directions for NER in a specific but concise manner. To prevent ChatGPT from rephrasing entities, we instructed it to extract their *exact* names from the input text. Task guidance provides entity definitions from the original RareDis annotation guidelines. The objective is to help ChatGPT differentiate between entity types within the context of the input text, as all four entities overlap semantically. Output specification instructs ChatGPT to output the extracted entities in a specific format to reduce post-processing workload. Output retrieval prompts the model to generate a response. In the few-shot setting, we also provided an example with an input text from the training set and its gold standard labels (i.e., entities labeled by the annotators).

*Prompt format*. In each setting, we experimented with two prompt formats: simple and structured (Table 2). The former presents the prompt as a simple sentence, and the latter a structured list. The simple sentence is shorter in length and resembles human instructions provided in a conversational setting where different building blocks (i.e., task instruction, task guidance, and output specification) are woven together as a single unit. Agrawal et al. [14] and Hu et al. [15] used a similar approach to extract medications and clinical entities, respectively. In contrast, the structured list resembles a recipe or outline that consists of multiple sub-prompts in a specific order. Chen et al. [16] used a similar format for evaluating ChatGPT’s NER performance on benchmark biomedical datasets. To provide additional guidance for ChatGPT, we also incorporated distinguishing characteristics about each entity in their prompts supplemented with examples (Table 3).

*Few-shot example selection.* We explored two strategies for selecting an example text in the few-shot setting. The first strategy involved randomly selecting a text from the training set, and the second selecting the training text that was most similar to the test text. The motivation for the second strategy was that different rare diseases may have similar etiology, course of progression, and symptoms/signs. For example, Creutzfeldt-Jakob disease and CARASIL (cerebral autosomal recessive arteriopathy with subcortical infarcts and leukoencephalopathy) are rare, neurological diseases that share similar signs, including progressive deterioration of cognitive processes and memory. Thus, providing a training text most similar to the test text may improve ChatGPT’s performance. To implement this strategy, we selected the training text with the highest similarity score based on spaCy’s pre-trained word embeddings and incorporated it as an example in the few-shot prompt [29]. We repeated this process for each text in the test set.

**Table 3:** Distinguishing characteristics of each entity

Entity	Distinguishing Characteristics
Rare disease	Rare diseases often come with terms like “rare", “uncommon", or mentions of specific low-prevalence numbers.
	For example, in the sentence, “Ablepharon-Macrostomia Syndrome (AMS) is an extremely rare inherited disorder,"
	“Ablepharon-Macrostomia Syndrome" and “AMS" are rare diseases, but “inherited disorder" is not.
Disease	Diseases are generally recognized medical conditions. The mention of a disease might not necessarily come with
	descriptors of its prevalence unless it’s rare. For example, in the sentence, "Ablepharon-Macrostomia Syndrome
	(AMS) is an extremely rare inherited disorder," "inherited disorder" is a disease.
Symptom	Symptoms are subjective and detected by the patient. For example, in the sentence, "In the acute form,
	drowsiness, coma, and seizures may occur," "drowsiness" is a symptom, but "coma" and "seizures" are not.
Sign	Signs can be measured or observed and don’t rely on the patient’s subjective reporting. For example,
	in the sentence, "In the acute form, drowsiness, coma, and seizures may occur," "coma" and "seizures" are signs,
	but "drowsiness" is not.

Figure 2b illustrates the architecture of the GPT model. In contrast to BERT, GPT uses a stack of transformer decoder layers aimed at autoregressive (left to right) text generation, i.e., predicting the next token based on preceding context. GPT takes as input a sequence of tokens for the prompt in addition to texts from the RareDis corpus and produces embeddings, which are then passed through decoder layers to produce contextualized representations. Unlike BERT, GPT does not use special tokens like CLS or SEP. Based on our prompts, the model directly outputs the predicted entities in a list separated by commas. Figure 2b shows an example where “Keratomalacia" and “corneal necrosis" were identified as rare disease entities. We performed post-processing to remove separating commas and, using the notation defined in Section 3.1, the predicted output vector in this example is $\hat{Y} = \{\hat{y}_1, \hat{y}_2\} = \{\{\text {Keratomalacia, rare disease}\}, \{\text {corneal necrosis, rare disease}\}\}.$

### Evaluation

#### Metrics

To evaluate model performance on the test set, we computed the following evaluation metrics: precision, recall, and F1-score. Precision $= \frac{\text {Number correctly predicted}}{\hat{m}}$ is the proportion of predicted entities found by the model that were correct, and recall $= \frac{\text {Number correctly predicted}}{m}$ the proportion of gold standard entities identified by the model. F1 $= \frac{2 \times \text {Precision} \times \text {Recall}}{\text {Precision} + \text {Recall}}$ accounts for both precision and recall by taking the harmonic mean. We calculated these metrics under two evaluation settings: exact and relaxed. For an exact match on the *j*th entity, the true and predicted entities must share the same boundaries and entity type, i.e., $x_{\text {start}_j} = x_{\hat{\text {start}}_j}, x_{\text {end}_j} = x_{\hat{\text {end}}_j}$ and $t_j = \hat{t}_j$. For a relaxed match, the predicted and true entity must overlap in their textual spans and have the same entity type, i.e., $\{x_{\text {start}_j}:x_{\text {end}_j}\} \cap \{x_{\hat{\text {start}}_j}:x_{\hat{\text {end}}_j}\} \ne \emptyset$ and $t_j = \hat{t}_j$. To ensure that stop words did not influence the evaluation, we removed them from both the gold standard and model-predicted entities.

#### Error Analysis

In our error analysis, we considered five types of errors: 1) incorrect boundary, 2) incorrect entity type, 3) incorrect boundary and entity type, 4) spurious, and 5) missed. The first refers to a predicted entity where one or both of its boundaries do not match that of the gold standard label, i.e., for entity *j*, $x_{\text {start}_j} \ne x_{\hat{\text {start}}_j}$, $x_{\text {end}_j} \ne x_{\hat{\text {end}}_j}$, or both. The second refers to a predicted entity with incorrect type, i.e., $t_j \ne \hat{t}_j.$ The third refers to the case where neither the predicted entity’s boundaries nor type matches those of the gold standard label. Spurious entities are predicted entities that do not correspond to any gold standard labels (false positive). In other words, predicted entity *k* is spurious if $\{x_{\hat{\text {start}}_k}:x_{\hat{\text {end}}_k}\} \cap \{x_{\text {start}_j}:x_{\text {end}_j}\} = \emptyset$ for all $j \le n$. Missed entities are true entities that the model failed to identify (false negative), i.e., entity *j* is missed if $\{x_{\hat{\text {start}}_k}:x_{\hat{\text {end}}_k}\} \cap \{x_{\text {start}_j}:x_{\text {end}_j}\} = \emptyset$ for all $k \le n$.

## Results

### Overall Results

*Fine-tuning vs. Prompt learning.* Table 4 provides a summary of the model performance by entity type. Under exact match, fine-tuning BioClinicalBERT resulted in F1-scores that ranged from 0.491 to 0.704, outperforming ChatGPT across all entity types. Under relaxed match, BioClinicalBERT achieved an overall F1-score of 0.689 and outperformed ChatGPT on all entities except rare diseases and signs. For these entities, prompt learning using ChatGPT in the few-shot setting resulted in higher F1-scores of 0.778 (vs. 0.755) and 0.725 (vs. 0.704) for rare diseases and signs, respectively. In the few-shot setting, ChatGPT outperformed BioClinicalBERT in terms of recall under relaxed match across all entity types.

**Table 4:** Summary of model performance by entity type

				Exact			Relaxed		
Paradigm	Model	Setting	Entity	Precision	Recall	F1	Precision	Recall	F1
Fine-tuning	BioClinicalBERT	Supervised	Rare disease	0.689	**0.720**	**0.704**	0.772	0.739	0.755
			Disease	**0**.**494**	**0**.**488**	**0**.**491**	**0.532**	0.538	**0**.**535**
			Sign	**0**.**561**	**0**.**516**	**0.538**	0.676	0.0735	0.704
			Symptom	**0**.**667**	0.630	**0**.**648**	**0.704**	0.745	**0**.**724**
			Overall	**0**.**600**	**0**.**583**	**0**.**591**	**0**.**681**	0.698	**0**.**689**
Prompt	ChatGPT	Zero-shot	Rare disease	0.559	0.409	0.472	0.843	0.694	0.761
learning		(Simple sentence)	Disease	0.109	0.240	0.150	0.200	0.437	0.274
			Sign	0.269	0.380	0.315	0.537	0.751	0.627
			Symptom	0.070	0.619	0.126	0.084	0.762	0.155
			Overall	0.203	0.369	0.262	0.365	0.670	0.472
		Zero-shot	Rare disease	0.765	0.489	0.597	0.887	0.634	0.740
		(Structured list)	Disease	0.184	0.210	0.196	0.261	0.293	0.276
			Sign	0.266	0.324	0.292	0.448	0.543	0.491
			Symptom	0.063	0.690	0.116	0.079	0.857	0.145
			Overall	0.226	0.359	0.277	0.331	0.528	0.407
		Zero-shot	Rare disease	0.663	0.613	0.637	0.821	0.763	0.791
		(Structured list	Disease	0.138	0.263	0.181	0.199	0.377	0.261
		+ Distinguishing	Sign	0.303	0.369	0.333	0.572	0.676	0.620
		Characteristics)	Symptom	0.068	0.643	0.123	0.086	0.810	0.156
			Overall	0.240	0.420	0.305	0.371	0.640	0.470
		Few-shot	Rare disease	0.719	0.441	0.547	0.937	0.634	0.756
		(Simple sentence	Disease	0.211	0.210	0.210	0.287	0.287	0.287
		+ Random example)	Sign	0.457	0.409	0.432	**0.721**	0.671	0.695
			Symptom	0.279	0.452	0.345	0.294	0.476	0.364
			Overall	0.423	0.376	0.398	0.616	0.568	0.591
		Few-shot	Rare disease	0.569	0.532	0.550	0.750	0.758	0.754
		(Structured list	Disease	0.151	0.341	0.209	0.211	0.467	0.291
		+ Random example)	Sign	0.273	0.406	0.327	0.478	0.698	0.567
			Symptom	0.094	**0**.**714**	0.166	0.107	0.810	0.189
			Overall	0.237	0.440	0.308	0.361	0.668	0.469
		Few-shot	Rare disease	0.677	0.608	0.640	0.812	0.769	0.790
		(Structured list	Disease	0.131	0.341	0.189	0.186	0.473	0.267
		+ Random example	Sign	0.268	0.366	0.310	0.539	0.743	0.625
		+ Distinguishing	Symptom	0.072	0.548	0.127	0.100	0.762	0.177
		Characteristics)	Overall	0.230	0.429	0.300	0.370	0.692	0.483
		Few-shot	Rare disease	**0**.**818**	0.484	0.608	**0.967**	0.634	0.766
		(Simple sentence	Disease	0.206	0.246	0.224	0.286	0.341	0.311
		+ Similar example)	Sign	0.441	0.444	0.443	0.720	0.730	**0**.**725**
			Symptom	0.260	0.310	0.283	0.308	0.381	0.340
			Overall	0.422	0.403	0.412	0.617	0.603	0.610
		Few-shot	Rare disease	0.590	0.565	0.577	0.762	**0**.**790**	0.776
		(Structured list	Disease	0.199	0.437	0.273	0.297	**0**.**653**	0.408
		+ Similar example)	Sign	0.337	0.487	0.398	0.561	0.802	0.660
			Symptom	0.093	0.690	0.164	0.114	**0**.**833**	0.200
			Overall	0.278	0.506	0.359	0.421	**0**.**769**	0.544
		Few-shot	Rare disease	0.596	0.586	0.591	0.766	0.790	**0**.**778**
		(Structured list	Disease	0.182	0.473	0.263	0.248	0.635	0.356
		+ Similar example	Sign	0.310	0.495	0.381	0.535	**0**.**818**	0.647
		+ Distinguishing	Symptom	0.076	0.619	0.135	0.091	0.738	0.162
		Characteristics)	Overall	0.257	0.519	0.343	0.385	0.767	0.513

Best scores by entity type are bolded

*Comparison across prompts.* Overall, incorporating an example in the few-shot setting led to improved performance over the zero-shot setting. Under relaxed match, ChatGPT in the zero-shot setting achieved F1-scores of 0.472 and 0.407 with the simple sentence and structured list prompts, respectively. Its performance improved in the few-shot setting, resulting in F1-scores of 0.591 and 0.469. Selecting a similar training text led to additional improvement, resulting in F1-scores of 0.610 and 0.544. Compared to prompts written as a structured list, simple sentences generally achieved similar or better performance; this trend was consistent across both zero- and few-shot settings. Incorporating distinguishing characteristics in the prompt led to an increase in the overall F1-score in the zero-shot (structured list) and few shot (structured list + random training text) settings. Moreover, this approach resulted in the highest F1-score for rare diseases (F1 = 0.778) in the few shot (structured list + similar training text) setting, outperforming BioClinicalBERT (F1 = 0.755).

*Comparison across entities.* Among the four entities, rare diseases were associated with the highest accuracy for both models across all settings. In contrast, diseases were challenging for both models. While BioClinicalBERT performed similarly at extracting signs and symptoms, ChatGPT achieved substantially better performance for signs. This trend was consistent across both zero- and few-shot settings.

### Detailed Error Analysis

We conducted an in-depth error analysis to elucidate ChatGPT’s performance. This analysis was crucial for gaining additional insight, as unlike other biomedical corpora, RareDis contains entities with overlapping semantics. Specifically, rare diseases are similar to diseases, and symptoms to signs. Depending on the context of the input text, diseases can also be symptoms or signs.

Table 5 shows the distribution of errors in the few-shot setting (simple sentence + random example) under exact match. The most common error type for rare diseases is false negative (45%) followed by incorrect entity type (31%). In the case of entity type errors, ChatGPT tended to label rare diseases as diseases. For diseases, signs, and symptoms, false positives and false negatives were the most common error types. Based on manual review, many of these errors can be attributed to the challenge of differentiating among these entities. Specifically, ChatGPT’s under-performance may be attributed to the challenge of inferring contextual meaning. For example, in the sentence, “a large percentage of primary antiphospholipid syndrome (APS) patients are women with recurrent pregnancy loss," the entity “recurrent pregnancy loss" was used to describe a population of women who have APS. However, ChatGPT mistakenly identified it as a sign of APS. Another challenge is differentiating between signs (observable and/or measurable) and symptoms (subjective to the patient/non-measurable). For example, ChatGPT mistakenly identified “weight loss" and “fever" as symptoms. In another example, it labeled “fatigue" as both a symptom and a sign, suggesting that it was challenging to for the model to understand the subtle difference between the two entities. In other cases, gold standard labels deviated from the definitions provided in the annotation guidelines, as the lack of abnormalities was also labeled as an entity (i.e., “asymptomatic during infancy or childhood" was labeled as a symptom by the annotators). As such, a portion of false negatives could be attributed to these edge cases.

**Table 5:** Error analysis for ChatGPT in the few-shot setting under exact match

	Boundary ✗	Boundary $\checkmark$	Boundary ✗	Spurious	Missed	Total
	Entity type $\checkmark$	Entity type ✗	Entity type ✗	(False Pos.)	(False Neg.)	errors
Rare disease	16 (10%)	48 (31%)	17 (11%)	4 (3%)	72 (45%)	157 (100%)
Disease	11 (4%)	7 (2%)	9 (3%)	147 (51%)	116 (40%)	290 (100%)
Sign	64 (17%)	8 (2%)	5 (1%)	146 (40%)	148 (40%)	371 (100%)
Symptom	3 (4%)	12 (16%)	2 (3%)	34 (44%)	25 (33%)	76 (100%)

## Discussion

In this work, we reformulated NER as a text generation task and established a benchmark for ChatGPT’s performance on extracting rare disease phenotypes. Overall, while fine-tuning BioClinicalBERT led to better performance, prompt learning using ChatGPT achieved similar or higher accuracy for some entities (i.e., rare diseases and signs) with a single example, demonstrating its potential for out-of-the-box NER in the few-shot setting. Given its accessibility, ChatGPT may be leveraged to extract rare diseases or signs without relying on a large, annotated corpus, which is a major bottleneck for training natural language processing models. Overall, prompts written as simple sentences generally achieved similar or better performance than structured lists, suggesting that ChatGPT may be more receptive to conversational prompts. To this end, we recommend using these prompts to identify and extract rare diseases and their phenotypes.

Our error analysis revealed that ChatGPT tended to label rare diseases as diseases. These errors may be attributed to the fact that there is no single definition of rare diseases; rather, the definition can vary by country or location (i.e., a disease is a rare disease if it affects $< 200,000$ people in the United States or no more than 1 in 2,000 in the European Union). Moreover, this definition is subject to change over time, as a disease that used to be rare at the time of annotation may have become more prevalent, or vice versa. Because annotations are contextual, it’s possible that what the domain experts deemed as rare diseases may not be reflected in information on the Internet before September 2021, ChatGPT’s knowledge cut-off date.

While other studies explored supervised deep learning techniques for extracting rare disease phenotypes, ours is the first to study ChatGPT in the zero- and few-shot settings. Segura-Bedmar et al. [21] compared the NER performance of base BERT, BioBERT, and ClinicalBERT, and found that ClinicalBERT had the highest overall F1-score (0.695). This was comparable to BioClinicalBERT’s performance in the current study (0.689). Fabregat et al. [26] used support vector machines and neural networks with a long short-term memory architecture to extract disabilities associated with rare diseases and obtained an F1-score of 0.81. While this was much higher than the overall F1-scores in the current study, the authors focused on extracting a single entity, i.e., disabilities, whereas our goal was to recognize and differentiate among four entities with overlapping semantics. Hu et al. [15] and Chen et al. [16] evaluated ChatGPT’s clinical and biomedical NER performance and found that it had lower accuracy than fine-tuning pre-trained LLMs. While our overall results aligned with this finding, we discovered that ChatGPT had similar or better performance on specific entities, suggesting that with appropriate prompt engineering, the model has the potential to match or outperform fine-tuned language models for certain entity types.

Our work has several potential limitations and extensions. First, we only had access to a subset of the RareDis corpus (832 out of 1041 texts), so our results may not fully reflect ChatGPT’s performance across the entire spectrum of rare diseases. Second, the current work focuses on ChatGPT and does not include GPT-4 or other variants (e.g., LLaMA, Alpaca, etc.), so broadening the current set of experiments to include other LLMs is a natural extension. Third, though manually-created prompts are highly intuitive and interpretable, evidence suggests that small changes can lead to variations in performance [30]. A promising alternative is to automate the prompt engineering process. To this end, Gutiérrez et al. [31] employed a semi-automated approach combining manually-created prompts with an automatic procedure to choose the best prompt combination with cross validation. In addition, fully-automated prompt learning approaches, where the prompt is described directly in the embedding space of the underlying language model, are also interesting extensions of the current work [32, 33]. Last, while the current study did not involve clinical data, prompt-learning strategies proposed herein are transferrable to clinical applications that leverage secure instances of ChatGPT. Specifically, these instances are governed by appropriate legal and business agreements ensuring privacy of protected health information. Given the ease of interacting with ChatGPT through textual prompts, our work has the potential to inform clinical applications on rare disease phenotyping in practice.

The advent of LLMs is creating unprecedented opportunities for rare disease phenotyping by automatically identifying and extracting disease-related concepts. While these models provide valuable insight and assistance, researchers and clinicians should critically evaluate model outputs and be well-informed of their limitations when considering them as tools for supporting rare disease diagnosis and treatment.

## Data Availability

The RareDis corpus can be found using the link provided in [19]. The code for the current study can be found at https://github.com/cathyshyr/rare_ disease_phenotype_extraction.
